# Plant uptake of phosphorus and nitrogen recycled from synthetic source-separated urine

**DOI:** 10.1007/s13280-014-0616-6

**Published:** 2015-02-15

**Authors:** Christophe Bonvin, Bastian Etter, Kai M. Udert, Emmanuel Frossard, Simone Nanzer, Federica Tamburini, Astrid Oberson

**Affiliations:** 1 Group of Plant Nutrition, Research Station Eschikon, Institute of Agricultural Sciences, ETH Zurich, Eschikon 33, 8315 Lindau, Switzerland; 2 Eawag, Swiss Federal Institute of Aquatic Science and Technology (PSI), 8600 Dübendorf, Switzerland; 3 Paul Scherrer Institut, 5232 Villigen, Switzerland

**Keywords:** Human urine, Struvite, Nitrified urine fertilizer, Phosphorus, ^33^P and ^15^N labeling, Nutrient use efficiency

## Abstract

Urine contains about 50 % of the phosphorus (P) and about 90 % of the nitrogen (N) excreted by humans and is therefore an interesting substrate for nutrient recovery. Source-separated urine can be used to precipitate struvite or, through a newly developed technology, nitrified urine fertilizer (NUF). In this study, we prepared ^33^P radioisotope- and stable ^15^N isotope-labeled synthetic NUF (SNUF) and struvite using synthetic urine and determined P and N uptake by greenhouse-grown ryegrass (*Lolium multiflorum* var. Gemini) fertilized with these products. The P and N in the urine-based fertilizers were as readily plant-available in a slightly acidic soil as the P and N in reference mineral fertilizers. The ryegrass crop recovered 26 % of P applied with both urine-based fertilizers and 72 and 75 % of N applied as struvite and SNUF, respectively. Thus, NUF and urine-derived struvite are valuable N and P recycling fertilizers.

**Electronic supplementary material:**

The online version of this article (doi:10.1007/s13280-014-0616-6) contains supplementary material, which is available to authorized users.

## Introduction

The growing world population and urbanization are resulting in increasing fluxes of phosphorus (P) and nitrogen (N) from agroecosystems to consumers, and eventually into wastewater. Globally, an estimated 3.4 Mt of P are excreted annually by humans, with about 50 % in urine (Mihelcic et al. 2011). If collected and recycled, the P contained in human excreta could satisfy one-fifth of the global P demand (Mihelcic et al. 2011). Nitrogen is mostly excreted in urine (about 90 %; Larsen and Gujer 1996), which could amount to about 28 Mt of human urine N per annum (rough estimate based on an N:P ratio in fresh urine of 16.5 (Udert et al. 2006) and a global annual total amount of urine P of 1.68 Mt (Mihelcic et al. 2011)). Separate collection and treatment of urine have been proposed as an alternative sanitation system that reduces environmental pollution and simplifies nutrient recovery (Larsen et al. 2013). Direct application of human urine as a fertilizer is common practice in many rural areas worldwide. However, this direct recycling pathway cannot be applied in modern cities for several reasons. For example, stored urine has a pungent smell and is prone to N losses due to ammonia volatilization; the high water content renders transport to cropping regions costly; and undesired constituents (e.g., pathogens, micro-pollutants such as synthetic hormones, pharmaceuticals, and their metabolites) pose a health risk to farmers and consumers (Larsen et al. 2013). New technologies are, therefore, needed to recover nutrients in hygienically safe products that can easily be transported to agricultural fields and that are available to plants.

Nearly all the P in urine is present as inorganic phosphate (Ciba-Geigy 1977). Struvite (MgNH_4_PO_4_·6H_2_O) precipitation is a proven technology to recover phosphate from wastewater and has also been applied successfully to recover P from source-separated urine (Etter et al. 2011). If magnesium (Mg) ions are dosed as Mg salts, nearly 100 % of the phosphate can be precipitated (Hug and Udert 2013). However, ammonium recovery in struvite may be only 5 % (Etter et al. 2011) and other valuable nutrients, such as potassium (K), sulfur (S), and micronutrients, are not recovered. A new technology therefore aims at complete nutrient recovery from urine. In a bioreactor, source-separated urine is stabilized by converting volatile ammonia into ammonium nitrate and by mineralizing 90 % of the organic carbon (C). In the second process step, most of the water is removed by distillation (Udert and Wächter 2012). The final product is a concentrated urine solution (nitrified urine fertilizer, NUF). As all the P, K, and S and more than 99 % of the N are recovered, NUF can be considered a multi-elemental fertilizer rich in N (210 mg g^−1^ dry matter; derived from Udert and Wächter, 2012) with potentially higher market value than struvite.

The state of research regarding P and N uptake by plants from struvite and NUF differs. The P uptake from struvite has been studied in the greenhouse with a variety of test plants on soils covering a range of pH from acidic to slightly alkaline and using synthetic struvite as well as struvite recovered from municipal, agricultural, or industrial wastewaters or from source-separated urine. Despite only 1–5 % of struvite-P being soluble in water (Cabeza et al. 2011; Achat et al. 2014b), all previous studies have found that struvite is as effective as water-soluble reference fertilizers in supplying P to plants (Johnston and Richards 2003; Massey et al. 2009; Antonini et al. 2011; Cabeza et al. 2011). However, apart from Achat et al. (2014b) who used a radioisotope approach in which the soil P was labeled with ^33^P, but not the fertilizer P, none of these studies differentiated the P taken up by plants into P derived from the soil and P derived from the fertilizer. These studies therefore do not allow accurate estimation of the amount of struvite-P taken up by plants. In addition, the effectiveness of the N contained in struvite has received little attention and simultaneous P and N uptake from struvite has not yet been studied. Moreover, due to its recent development, NUF has not yet been tested as a plant fertilizer.

The aim of the present study was to determine uptake by ryegrass growing in pots in a greenhouse of P and N from ^33^P- and ^15^N-labeled struvite (STR) and synthetic-nitrified urine fertilizer (SNUF) produced from synthetic urine as a model for source-separated human urine. The starting hypothesis was that SNUF and STR can supply ryegrass with P and N as effectively as water-soluble mineral P and N fertilizers.

## Materials and methods

### Labeled synthetic urine-based fertilizer production

The nutrient composition of stored source-separated human urine can be mimicked by synthetic urine (Wilsenach et al. 2007; Tilley et al. 2008), which has been used, e.g., for studying the thermodynamics of struvite precipitation (Ronteltap et al. 2007). Consequently, the effectiveness of human urine P and N can be studied by applying isotope labeling to synthetic urine. In the present study, labeling with ^15^N and ^33^P took place during preparation of the synthetic urine solutions.

For STR production, a synthetic urine solution was prepared based on Udert et al. (2006) (Table 1). The biodegradable substrate (80 % of total chemical oxygen demand, COD) was mimicked with acetate. Monosodium phosphate (NaH_2_PO_4_·H_2_O) was first added to deionized water. Thereafter, the equivalent of 27 MBq ^33^P radioactivity per liter was added in the form of carrier-free ^33^PO_4_^3−^ (^33^P-phosphoric acid; Hartmann Analytics GmbH, Braunschweig, Germany). After 10 min stirring, all other urine components (Table 1) were dissolved one by one into the ^33^P-monosodium phosphate solution. The solution was ^15^N-enriched by adding ^15^N-ammonium acetate (^15^NH_4_C_2_H_3_O_2_, 98 at.% ^15^N; Sigma Aldrich Chemie GmbH, Buchs, Switzerland) in amounts to achieve 3.2 at.% ^15^N excess in the solution (excess over the 0.3663 at.% ^15^N of atmospheric N_2_) (Table 2). Prior to the precipitation of STR, the pH of the synthetic urine solution was checked (8.9) and Mg (MgCl_2_) was added at a molar Mg:P ratio of 1.5:1 to ensure that all the P precipitated (Wilsenach et al. 2007). The final solution was stirred for 75 min and then filtered at 500 mbar through cellulose filters (0.45 μm; Whatman, Maidstone, UK). The recovered STR was dried in vacuum desiccators and the STR granules were homogenized into a fine powder with a pestle and mortar and stored in desiccators until use.

**Table 1:** Composition of labeled synthetic urine for struvite (STR) production and of labeled synthetic-nitrified urine for synthetic-nitrified urine fertilizer (SNUF) production, including concentrations of chemicals (mM) and compounds (g L^−1^). Adapted from Udert et al. (2006) for synthetic source-separated stored urine and from Udert and Wächter (2012) for synthetic source-separated nitrified stored urine. TIC = total inorganic carbon, COD = chemical oxygen demand

Synthetic urine	STR	NH_3_-N	NO_3_-N	PO_4_-P	TIC	SO_4_	K	Na	Cl	COD
	mM	g L^−1^								
NaH_2_PO_4_·H_2_O	17	–	–	0.54	–	–	–	0.40	–	–
NH_4_-acetate	104	1.45	–	–	–	–	–	–	–	6.64
^15^NH_4_-acetate (98 at.% ^15^N)	21	0.29	–	–	–	–	–	–	–	1.33
NH_4_OH (solution 28% NH_3_)	175	2.45	–	–	–	–	–	–	–	–
NH_4_HCO_3_	271	3.79	–	–	3.25	–	–	–	–	–
Na_2_SO_4_ anhydrous	16	–	–	–	–	1.56	–	0.74	–	–
NaCl	62	–	–	–	–	–	–	1.43	2.21	–
KCl	56	–	–	–	–	–	2.19	–	1.99	–
Total		7.98	0	0.54	3.25	1.56	2.19	2.58	4.19	7.97

Synthetic nitrified urine	SNUF	NH_3_-N	NO_3_-N	PO_4_-P	TIC	SO_4_	K	Na	Cl	COD
	mM	g L^−1^								
NaH_2_PO_4_·H_2_O	17	–	–	0.54	–	–	–	0.39	–	–
NH_4_NO_3_	155	2.17	2.17	–	–	–	–	–	–	–
^15^NH_4_^15^NO_3_ (10 at.% ^15^N)	85	1.19	1.19	–	–	–	–	–	–	–
Na_2_SO_4_ anhydrous	16	–	–	–	–	1.54	–	0.74	–	–
NaCl	62	–	–	–	–	–	–	1.43	2.20	–
KCl	57	–	–	–	–	–	2.23	–	2.02	–
HCl 37 %	0.005	–	–	–	–	–	–	–	<0.001	–
Total		3.36	3.36	0.54	0	1.54	2.23	2.55	4.22	0

**Table 2:** Form applied, dry matter (DM) content, total P and N concentration, isotopic composition (^15^N atomic enrichment, specific activity (SA)) and solubility in water of the fertilizers used in the study. STR = struvite, SNUF = synthetic-nitrified urine fertilizer

Fertilizer	DM content	Total N	Total P	^15^N atomic enrich	*SA*	Solubility of elements in H_2_O^a^	Solubility of elements in H_2_O^a^
	%	mg g^−1^	mg g^−1^	% ^15^N	Bq µg^−1^ P	% of total N	% of total P
^15^NH_4_^15^NO_3_	–	340^b^	0	0.6	n.a	100	100
KH_2_^33^PO_4_	–	0	227^b^	n.a.	29.2	100	100
Labeled STR	90	53	132	3.2	35.1	3	1.8
Labeled SNUF	97	218	17	3.5	32.4	97	88.9

^a^Water-soluble phosphate, ammonia, and nitrate ions determined in product suspensions (2.5 g fertilizer per 250 mL water). Method from European Parliament (2003)

^b^Refers to the concentration of the salt applied

The composition of the synthetic-nitrified urine solution used for SNUF production was based on Udert and Wächter (2012). No bicarbonate was added to the synthetic nitrified urine, because the bicarbonate concentration after nitrification is very low. Monosodium phosphate was first dissolved in deionized water, followed by the addition of 27 MBq ^33^P radioactivity per liter in the form of carrier-free ^33^PO_4_^3−^ (^33^P-phosphoric acid; Hartmann Analytics GmbH). After 10 min stirring, all other urine components (Table 1) were dissolved one by one into the ^33^P-monosodium phosphate solution. The nitrified urine solution was spiked with ^15^N-ammonium nitrate (^15^NH_4_^15^NO_3_, 10 at.% ^15^N; Sigma Aldrich Chemie GmbH) in amounts to achieve ^15^N enrichment of 3.2 at.% ^15^N excess in the solution (Table 2). The pH of the synthetic-nitrified urine solution was adjusted with hydrochloric acid (1 M) to match the pH (6) of urine effluent from the nitrification reactor (Udert and Wächter 2012). The distillation of synthetic-nitrified urine was carried out in order to recover a maximum of nutrients in the form of solid SNUF. A volume of 200 mL synthetic-nitrified urine was distilled at once at low pressure using a Büchi Rotavap (for complete distillation protocol, see Udert and Wächter 2012). The solid SNUF was also homogenized into a fine powder and then stored in a vacuum desiccator until application.

### Growth experiment

To assess the P and N uptake from SNUF and STR, a pot experiment with Italian ryegrass (*Lolium multiflorum* var. Gemini) was carried out. The soil used for the growth experiment was taken from the 0–20 cm layer of an arable field in Heitenried (Freiburg, Switzerland), where long-term cropping has been practiced. This acidic soil (pH in water 5.4) was selected because its moderate P availability results in a growth and P uptake response to P fertilizer inputs (Nanzer et al. 2014). It is classified as a Mollic Fluvisol (FAO classification), and has a sandy loam texture (16 % clay, 26 % silt, and 58 % sand), 14 g kg^−1^ of total C and 1.3 g kg^−1^ of total N and a low cation exchange capacity (CEC 21 cmol^+^ kg^−1^) (Nanzer et al. 2014). Moist soil samples were sieved at 5 mm, then stored until use. The equivalent of 1 kg soil dry matter (DM) was used per pot.

The growth experiment comprised seven treatments (five controls and two urine-based fertilizer treatments) in a completely randomized design with four replicates, resulting in a total of 28 pots (Table 3). Treatments consisted of (1) unfertilized control (0N0P), (2) N-fertilized control (1N0P), (3) P-fertilized control (0N1P), (4 and 5) reference control fertilizers (1N1P and 1N1.5P), and (6 and 7) two urine-based fertilizer treatments (STR and SNUF). For all fertilized control treatments except 1N1.5P, P and N plant uptake were studied using ^15^N- and ^33^P-labeled fertilizers (Table 2). The ^15^N-labeled reference fertilizer was produced using ^15^NH_4_^15^NO_3_ (10 at.% ^15^N; Sigma Aldrich Chemie GmbH), which was mixed with unlabeled NH_4_NO_3_ so as to reach the excess of 0.6 at.% ^15^N (Table 2). The ^33^P-labeled reference fertilizer was produced by mixing 30 MBq of carrier-free ^33^PO_4_^3−^ (^33^P-phosphoric acid; Hartmann Analytics GmbH) with KH_2_PO_4_ (Table 2). The synthetic urine-based fertilizer treatments consisted of the double ^33^P- and ^15^N-labeled STR and double-labeled SNUF treatments. The reference 1P dose was defined at 50 mg P kg^−1^ soil and the 1N dose at 100 mg N kg^−1^ soil, based on the plant requirements for intensively managed grassland (Flisch et al. 2009). Assuming a bulk density of 1.3 t m^−3^ and distribution of the fertilizers within the 0–5 cm soil layer, this would correspond to in kg ha^−1^, 33 P and 65 N. It was also expected to be within the range of agronomic plant response (Barrow 1985), which was confirmed by response curves (Electronic Supplementary Material, Figs. S1, S2). The urine-based fertilizers were applied on the day of ryegrass sowing. Given its N:P mass ratio, STR with N:P of 1:2.2 supplied the full dose of P (50 mg P kg^−1^ soil) and adjustments with non ^15^N-labeled NH_4_NO_3_ were made to reach the level of 100 mg N kg^−1^ soil (Table 3). In contrast, SNUF with N:P of 9.5:1 supplied the entire N dose but needed complementary P in the form of unlabeled KH_2_PO_4_ (Table 3). Likewise, the control treatments were P- and N-fertilized on the day of sowing, as indicated in Table 3. For both STR and SNUF, 1N1P was considered the reference (control) treatment. Because of a problem with the N amendment of 1N1P (0.6N added instead of 1N, see footnote of Table 3), 1N1.5P was also used as the control for N. All macronutrients other than P and N were fertilized at rates exceeding 2–3 times the expected plant nutrient uptake. At sowing, all treatments received 200 mg K, 60 mg Ca, 50 mg Mg, and 74 mg S per kg^−1^ soil DM and micronutrients. In parallel to the main growth experiment, a response curve experiment was carried out in which plant response to graduated amendment levels of P (0–150 mg kg^−1^ soil DM) and N (0–300 mg kg^−1^ soil DM) was investigated (Figs. S1, S2). For both the growth experiment and response curve experiment, 1 g of Italian ryegrass seeds was sown per pot. The pots were watered (deionized water) daily up to 50 % of water-holding capacity (560 g kg^−1^) during the first week, then up to 60 % during the remainder of the growth experiment. The base of the pots was sealed. The growth experiment took place in a greenhouse under controlled climate conditions: 85 % air humidity during the first 2 weeks, then 65 %, photoperiod 12 h day^−1^, temperature 22°C during day, and 18°C at night and light intensity of maximum ≈500 μmol m^−2^ s^−1^ artificial light. Pots were fully randomized three times per week. The ryegrass was harvested 30, 51, and 72 days after sowing. Plants were cut 3 cm above the soil surface at the first and second cut, and at 0.5 cm above the soil surface for the final cut. As the main focus was on P, the growth experiment was designed to provoke a high plant demand for that element and therefore P was not re-applied during the experiment. However, to exclude other nutrient limitations, all treatments were re-fertilized with unlabeled N (NH_4_NO_3_), K, Ca, and Mg after the first and second cut, at rates matching those received at the beginning of the experiment. Aboveground plant material was dried for 72 h at 50°C and then finely milled (sieve <2 mm). For each harvest, aboveground plant DM yield, P and N concentrations, and the isotopic composition (at.% ^15^N and specific activity (SA)) of plant material were determined.

**Table 3:** Rate and form of P and N applied at the beginning of the pot experiment and after each cut for all treatments

Treatment	Urine-based fertilizer		Water-soluble fertilizer		After cut 1 and 2
	N dose	P dose	N dose	P dose	N dose
	mg element kg^−1^ soil				
*Reference fertilizers* ^c^					
0N0P	0	0	0	0	0
0N1P^a^	0	0	0	50^a^	0
1N^b^0P	0	0	100^b^	0	100
1N^b^1P^a^	0	0	60^b,d^	50^a^	100
1N1.5P^e^	0	0	100	75	100
*Urine-based fertilizers*					
STR^a,b^	23^b^	50^a^	77	0	100
SNUF^a,b^	100^b^	8^a^	0	42	100

^a^Treatments labeled with ^33^P

^b^Treatments labeled with ^15^N

^c^Control treatments named according to P and N rates applied at sowing with respect to reference amounts

^d^1N1P abbreviation was kept despite the lower initial applied dose of 60 instead of the reference 100 mg N kg^−1^ soil foreseen at sowing. The distorted initial amount had no influence on total P_uptake_ (cumulative ${\text{P}}_{{{\text{uptake}}_{{ 1 {\text{N1P}}}} }} = {\text{P}}_{{{\text{uptake}}_{{ 1 {\text{N1}} {\cdot} 5 {\text{P}}}} }}$) or on cumulative DM production (DM_1N1P_ = DM_1N1.5P_). As N was re-applied after each cut (above table), 1N1P received only 13 % less N than other N-fertilized treatments over the course of the growth experiment

^e^Fertilized with unlabeled NH_4_NO_3_ and KH_2_PO_4_

### Fertilizer analyses

Total P and N concentrations in STR and SNUF were determined by wet chemical extraction. Subsamples of 25 mg fertilizer DM were dissolved in 100 mL 1 M HCl. Phosphorus concentrations were determined in the diluted extracts by colorimetry (Ohno and Zibilske 1991). Total N in the extract was measured on a TOC/TN analyzer (Skalar Formacs^HT/TN^, The Netherlands) and nitrate–N and ammonium-N with a continuous flow injection analyzer (Skalar San^++^ System, The Netherlands). Extracts were prepared in triplicate.

Water-soluble P and N content in STR and SNUF were determined in 1:100 suspensions (2.5 g fertilizer:250 mL deionized water). The suspensions were placed for 30 min on a rotary shaker at 40 rpm, filtered (0.45 µm) through a cellulose filter (Whatman, Maidstone, UK), and soluble P concentrations in diluted filtrates were determined by colorimetry (Ohno and Zibilske 1991). The soluble N concentrations were determined with a TOC–TN analyzer (IL 550 TOC–TN, Hach-Lange, Berlin, Germany). The crystal structure of STR was compared with that of struvite recovered from stored real human urine by X-ray powder diffractometry (XRD) (X’Pert^3^ Powder, PANalytical, Almelo, The Netherlands). For total N and ^15^N abundance measurements, SNUF (0.2 mg) and STR (1 mg) subsamples were analyzed on an elemental analyzer (Vario PYRO cube in combustion mode, Elementar, Hanau, Germany) coupled to a mass spectrometer (IsoPrime100, isotope ratio mass spectrometer, Manchester, UK).

### Plant analyses

Plant DM samples of 400 mg were ashed for 3 h at 550°C in a muffle oven. The ash was extracted with 2 mL 65 % HNO_3_ on a hot stirring plate, filtered through a 0.2 µm cellulose acetate syringe filter (Minisart NML, Sartorius, Göttingen, Germany), and P concentration was determined by colorimetry (Ohno and Zibilske 1991). The ^33^P beta emissions in fertilizer and plant extracts were measured by liquid scintillation counting (TRI-CARB 2500TR Liquid Scintillation Analyzer, Packard Instruments, Meriden, USA). Checks of the measurements revealed no quenching due to matrix effects. The values were corrected for radioactive decay to the day of planting (15 August 2013).

### Calculation of P and N uptake from fertilizer

The proportion of plant P originating from fertilizer (Pdff%) can be calculated from the SA in plants grown on soil fertilized with labeled ^33^P fertilizers (Morel and Fardeau 1990):

1 $$ {\text{Pdff}}\% = \frac{{\text{SA}_{\text{plant}} }}{{\text{SA}_{\text{fertilizer}} }} \times 100, $$

where SA_plant_ is the SA (^33^P/^31^P, Bq µg^−1^ P) in plants fertilized with ^33^P-labeled fertilizer and SA_fertilizer_ is the SA of the respective fertilizer. The amount of fertilizer P taken up by the plants (Pdffmg, mg P kg^−1^ soil) is

2 $$ {\text{Pdffmg}} = \frac{{{\text{Pdff}}\% \times \text{P}_{\text{uptake}} }}{100}, $$

where P_uptake_ is the P taken up by the plants (mg P kg^−1^ soil), calculated as follows:

3 $$ {\text{P}}_{\text{uptake}} = \, {\text{P}}_{\text{conc}} \times {\text{DM shoot yield,}} $$

where P_conc_ is the P concentration in shoots (mg P g^−1^ plant DM) and DM shoot yield is given in g kg^−1^ soil, which equals the yield per pot. The Pdff% was calculated for each of the three cuts and the resulting Pdff mg values were added together.

The P fertilizer recovery in plants (FertPrec, %) can be derived as follows:

4 $$ {\text{FertPrec}} = \frac{\text{Pdffmg}}{{{\text{P}}_{\text{applied}} }} \times 100. $$

The relative agronomic effectiveness (RAE, %) of STR was calculated by comparing its Pdffmg value to that of the reference fertilizer added at the same fertilization rate (Morel and Fardeau 1990; Nanzer et al. 2014):

5 $$ {\text{RAE}} = \frac{{{\text{Pdffmg}}_{\text{STR}} }}{{{\text{Pdffmg}}_{{1{\text{N}}1{\text{P}}}} }} \times 100. $$

For SNUF, the RAE could not be calculated as it was added at a lower dose than with 1N1P. Phosphorus derived from the soil (Pdfs, mg P kg^−1^ soil) was calculated as follows:

6 $$ {\text{Pdfs}}_{{}} = \, {\text{P}}_{\text{uptake}} - {\text{Pdffmg}} . $$

Analogously to P, the proportion of plant N originating from fertilizer (Ndff%, %) was calculated based on isotopic dilution principles (Barraclough 1995):

7 $$ {\text{Ndff}}\% = \frac{{^{15} {\text{N}}_{\text{excess \,plant}} }}{{^{15}{\text{N}}_{\text{excess \,fertilizer}} }}, $$

where ^15^N_excess plant_ and ^15^N_excess fertilizer_ are the ^15^N enrichments (at.% ^15^N excess) in the plant and in the fertilizer, respectively. The ^15^N enrichment of plant samples was calculated by subtracting the ^15^N abundance in the non N-fertilized plant samples, and the ^15^N excess of the fertilizers was calculated by subtracting the ^15^N excess in unlabeled fertilizers.

The total N taken up by plants (N_uptake_, mg N kg^−1^ soil), the amount of N derived from the fertilizer (Ndffmg, mg N kg^−1^ soil) and from the soil (Ndfs, mg N kg^−1^ soil), and the fertilizer N recovery in plants (FertNrec, %) were calculated using similar equations as for P (Eqs. 1–4, 6).

The Pdfs and Ndfs values included the P and N contained in the ryegrass seeds. The 1 g seed sown in the pots contained on average 3.1 mg P (Nanzer et al. 2014) and an estimated 20 mg N (Flisch et al. 2009).

### Statistical analyses

Statistical analyses were performed with the statistical software package R 2.15.1 (R Core Team 2013). A one-way analysis of variance was run to test the significance of the fertilizer treatments. The Shapiro–Wilk test was performed to test the residuals for normal distribution, followed by a graphical verification by means of quantile–quantile plots. Multiple comparisons were made using the Tukey HSD test (*α* = 0.01).

## Results

### Characteristics of processed urine-based fertilizers

All ^33^P-labeled fertilizers (STR, SNUF, and the KH_2_PO_4_ used for the 1P reference water-soluble P fertilizer) had similar SA at the beginning of the pot experiment (Table 2). The urine-based fertilizers had similar ^15^N enrichment, around 3 at.% ^15^N, while that in the mineral reference fertilizer was lower. Only small proportions of the total P and N in STR were soluble in water (2–3 % for both), but the P and N contained in SNUF were almost entirely water soluble (Table 2).

### Plant dry matter production

**Fig. 1:**
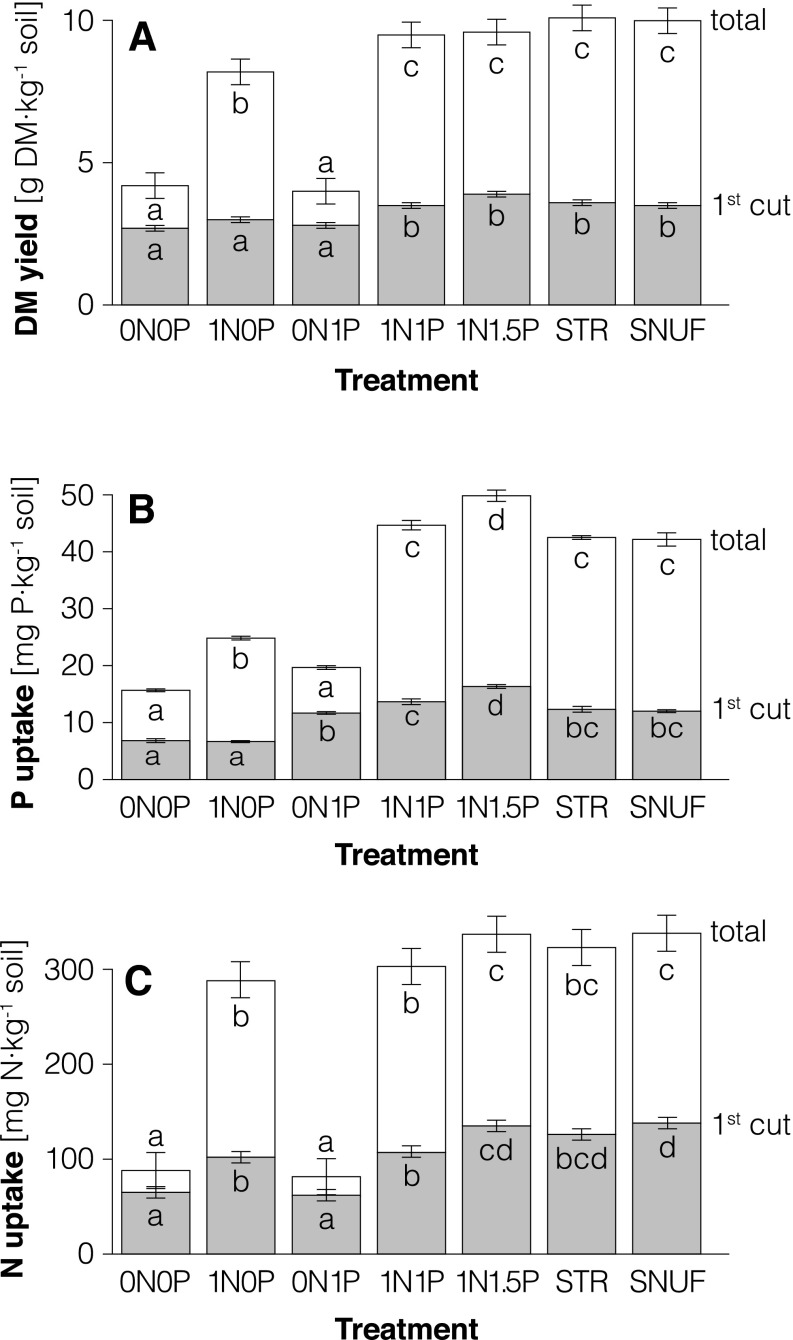
**a** Dry matter (DM) yield at the first cut of Italian ryegrass shoots and cumulative yield over all three cuts (total); **b** P_uptake_ at first cut and in total; and **c ** N_uptake_ at the first cut and in total. *STR* struvite, *SNUF* synthetic-nitrified urine fertilizer. *Vertical* *bars* indicate standard deviations

Cumulative shoot DM yield of Italian ryegrass varied between 4 and 10 g kg^−1^ soil. A significant yield response (*p* < 0.05) to fertilizer treatments was obtained from the first cut onwards and throughout the two remaining cuts (Fig. 1a). The treatments receiving no fertilizer (0N0P) and those receiving either no N or no P (0N1P and 1N0P) produced lower DM yield at the first cut and lower cumulative DM yield than the urine-based fertilizer or 1N1P and 1N1.5P control treatments. The STR and SNUF treatments produced the highest cumulative DM yield, but yield did not differ significantly from that obtained with the 1N1.5P and 1N1P treatments.

### Total P and fertilizer P uptake by plants

There was a strong response to water-soluble P fertilizers, as increasing doses resulted in significantly higher plant P uptake values (Fig. S1). Plant P uptake showed a linear response (*R* ^2^ = 0.99) to P amendment up to a fertilization rate of 50 mg P kg^−1^ soil.

**Fig. 2:**
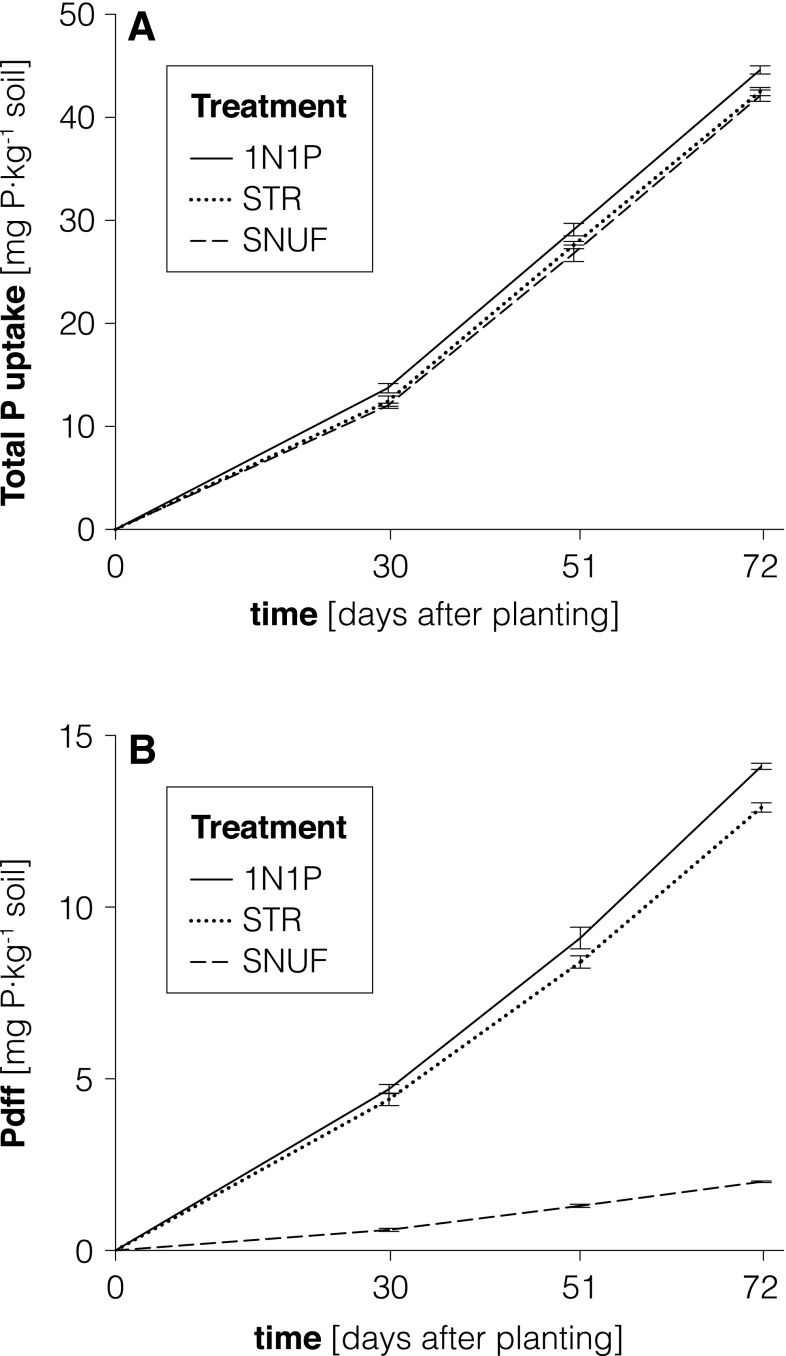
**a** Cumulative P uptake by ryegrass shoots and **b** shoot P uptake originating from fertilizer. (Pdffmg) in the 1N1P, STR and SNUF treatments, determined at 30, 51 and 72 days after planting. *STR* struvite, *SNUF* synthetic-nitrified urine fertilizer. *Vertical bars* indicate standard deviations

All P- and N-fertilized treatments had higher average shoot P concentrations (4.4–5 mg P g^−1^ DM) than the 1N0P treatment (3.1 mg P g^−1^ DM). This, together with the higher DM yield, resulted in significantly higher P uptake (Fig. 1b). Total P uptake in the 1N1P, STR, and SNUF treatments was similar (42–45 mg P kg^−1^ soil). The contribution of fertilizer P (Pdff%) to the total plant P uptake was around 30 % for the 1N1P and STR treatments (Table 4). For SNUF, where less P was added with the labeled fertilizer (Table 3), 5 % of total plant P originated from the SNUF. The three cuts of the ryegrass allowed the availability of fertilizer P to be determined at three time points. It was found that plants fertilized with STR and with reference water-soluble P (1N1P) took up the same amount of fertilizer P (Pdffmg) in all three growth periods. Plants in the STR and 1N1P treatments displayed similar fertilizer P uptake patterns (Fig. 2b). For both treatments, the proportion of fertilizer P in the plant (Pdff%) was higher during the first growing period, while total P uptake increased before the second and third cut (Fig. 2a). The Pdff values for plants in the SNUF treatment remained constant in absolute terms throughout the growth experiment (Pdffmg, Fig. 2b) and relative to total plant P uptake (Pdff%, 5 %), although at a lower level than with 1N1P and STR. The agronomic effectiveness relative to water-soluble P (RAE) of STR was 97 %. In terms of applied fertilizer P recovered in the plants (FertPrec), plants in the SNUF, STR, and 1N1P treatments recovered a similar proportion (26–28 %). The average cumulative Pdfs did not differ between the STR and 1N1P treatments, as the soil delivered an equivalent amount (30 mg P kg^−1^ soil) to both treatments. The Pdfs for the SNUF treatment could not be assessed, as the soil was fertilized with two P sources (SNUF and KH_2_PO_4_).

**Table 4:** P and N recovered in plant shoots of Italian ryegrass derived from different sources

Treatment	Pdfs	Pdff		FertPrec		Relative effectiveness	Ndfs	Ndff			FertNrec	
	All 3 cuts						1st cut					All 3 cuts
	mg P kg^−1^ soil	mg P kg^−1^ soil	%	%		%	mg N kg^−1^ soil	mg N kg^−1^ soil	%		%	
1N1P	30	14b	32b	28		n.a.	82	36b	34b		59	77
STR	30	13b	30b	26		97	n.a.	13a	10a		57	72
SNUF	n.a.	2a	5a	26		n.a.	84	57c	42c		57	75
SEM	0.4	3.3	7.6	0.7		n.a.	1.4	5	4		0.3	0.8
*ANOVA source of variation*												
Treatment	n.s.	***	***	n.s.		n.a.	n.s.	***	***		n.s.	n.s.

Parameters included P derived from soil (Pdfs), from fertilizer (Pdff) and fertilizer P recovery (FertPrec) in total for three cuts. N derived from soil (Ndfs) at first cut and from fertilizer (Ndff) at first cut, fertilizer N recovery (FertNrec) at first cut and in total, and agronomic effectiveness relative to water-soluble P (RAE, %) of struvite (STR) in total for the three cuts

Letters indicate significant differences between fertilizers (Tukey’s HSD, *α* = 0.01)

*n.s.* not significant, *n.a.* not applicable, *SEM* standard error of the mean

*, **, *** Significant at *p* < 0.05, *p* < 0.01 and *p* < 0.001 probability level, respectively

### Total N and fertilizer N uptake by plants

There was a strong response to N, with increased application of water-soluble N resulting in higher plant N uptake (linear response (*R* ^2^ = 0.99) up to 150 mg N kg^−1^ soil; Fig. S2). Thus as for P, the reference fertilization rate of 100 mg N kg^−1^ soil was in the linear response range.

Nitrogen fertilization resulted in higher N shoot concentrations and hence higher N uptake (Fig. 1c). The shoot N concentration in the 0N1P treatment (14 mg N kg^−1^) was significantly lower (*p* < 0.01) than in all N-fertilized treatments (range 28–30 mg N g^−1^ DM). Nitrogen uptake and Ndff were evaluated primarily for the first cut, as unlabeled NH_4_NO_3_ was added after each cut. In the SNUF treatment, at the first cut, over 40 % of plant N taken up originated from the fertilizer (Ndff%) (Table 4). For the 1N1P treatment, fertilizer N accounted for 34 % of plant N uptake (Ndff%), whereas it contributed a smaller proportion in the STR treatment (10 %). The low Ndff% of STR was expected, given the low initial dose (22 mg N kg^−1^ soil) (Table 3). The fertilizer N recovery pattern was comparable in all three treatments in terms of partial recovery at each cut and total recovery (Table 4). Thus at the first cut, close to 60 % of applied N was recovered in plants (Table 4), at the second cut between 10 and 12 % was recovered, and at the third cut plants recovered between 5 and 7 % of initially applied N. In total, FertNrec ranged between 72 and 77 % for STR, SNUF, and 1N1P (Table 4). The SNUF and 1N1P treatments withdrew similar amounts of N from the soil, resulting in Ndfs values of between 82 and 84 mg N kg^−1^ soil at the first cut (Table 4). As the STR treatment included two N fertilizers (STR and NH_4_NO_3_), Ndfs could not be assessed for that treatment. For all three treatments (1N1P, STR, SNUF), the majority (78–80 %) of total fertilizer N uptake occurred during the first 30 days of growth. The remaining 18–22 % of total fertilizer N uptake took place during the remaining 42 days of the growth experiment (data not shown).

## Discussion

### Properties of urine-based fertilizers

The water solubility of P and N in STR, which was prepared from artificial urine, was comparable to that of the P and N in struvite precipitated from real human urine (Antonini et al. 2012; Electronic Supplementary Material, Table S1). Likewise, the XRD results suggested similar elemental composition and crystal structures of STR and struvite from real human urine (Electronic Supplementary Material, Fig. S3).

The expected high water solubility of N in SNUF (97 %) was similar to the solubility of N in NUF produced from real human urine (93 % of total N in internal reference NUF), while the P solubility (89 %) of SNUF was higher than the P solubility in the internal reference NUF (57 %) (Table 2, Table S1). The procedure used for SNUF production was based on the chemical composition reported for nitrified human urine (Udert and Wächter 2012), so the differences in P solubility in the final solid NUF products may have been caused by differences in the distillation and drying process used for SNUF and internal reference NUF. Optimization of this process is still in progress and our preliminary findings suggest that it can affect the solubility of P.

### Effectiveness of P from urine-based fertilizers

Graduated application of mineral P resulted in increasing plant P uptake (Fig. S1), confirming earlier results obtained on the same soil (Nanzer et al. 2014). The P response curve also showed that the reference dose of 50 mg P kg^−1^ was in the linear response range, where fertilizer P is used most efficiently. In addition, the linear response in the P amendment treatments ranged from 0 to 50 mg P kg^−1^ soil, which allowed comparison of plant P recovery (FertPrec) even at the lower ^33^P-labeled P dose applied with SNUF (8.3 mg P of the total 50 mg P kg^−1^ soil; Table 3). There was a significantly higher P concentration (*p* < 0.05) and significantly higher P uptake (*p* < 0.05) for plants receiving the reference P fertilization dose (1N1P and STR) than plants receiving no P (1N0P) at all three cuts. This indicated that the P fertilizers were plant-available throughout the growth experiment and that P was a growth-limiting factor for the 1N0P treatment. As confirmation, the P concentration (2.2 mg P g^−1^ DM) at the first cut of the 1N0P treatment was below the critical concentration of 2.5 mg P g^−1^ DM reported for ryegrass (Bailey et al., 1997). In contrast, the P concentration (3.4–4.2 mg P g^−1^ DM) exceeded the threshold for all P-fertilized treatments, indicating sufficient P supply for the ryegrass plants.

The similar proportions and amounts of Pdff for STR and the water-soluble P reference fertilizer (1N1P) accumulated over the entire growth experiment (Table 4) indicate the high effectiveness of P contained in STR. The plant availability of P contained in different struvite products has been extensively studied and confirmed to be effective on a wide range of soils (Massey et al. 2009; Cabeza et al. 2011; Antonini et al. 2012). Using ^33^P isotopic dilution, Achat et al. (2014b) found an effectiveness (RAE) for synthetic struvite of 102–115 % on a slightly acidic soil (pH 6.5) with three cuts of a mixture of ryegrass and fescue and an initial amendment of 50 mg P kg^−1^ soil, which was similar to our RAE value (97 %; Table 4). Future studies should address the effectiveness of residual P from STR in the soil and compare it with the effectiveness of the residual P from water-soluble reference fertilizers and other recycling products.

Struvite is considered to be a slow-release P fertilizer (Massey et al. 2009; Rahman et al. 2011), given its low solubility in water (Cabeza et al. 2011; Table 2). Its solubility is also affected by pH, as shown, e.g., in a batch experiment with buffered solutions, where the solubility of struvite-P at different pH values (5.9, 7.0, and 8) was less than for triple superphosphate-P (Massey et al. 2009). In the present study, the highest Pdff% value (35 %) for the STR treatment was recorded at the first cut and was equal to that of the water-soluble mineral P, suggesting that P from STR solubilized rapidly in the soil solution. Homogeneous mixing of finely ground STR into the soil, sufficient daily watering (Lunt et al. 1964), and the acidic character of the soil (Babic-Ivancic et al. 2002; Doyle and Parsons 2002) probably had a positive effect on the dissolution rate of the STR crystals. Thus, our results confirm findings by Cabeza et al. (2011) and Achat et al. (2014a) that the effectiveness of recycled P products such as struvite is more accurately indicated by their dissolution in soil than by solubility tests in batch experiments. The use of ^33^P-labeled struvite provides a means to study the release kinetics and describe the fate of struvite or NUF products in the matrix of soils with differing properties.

Total fertilizer P recovery in ryegrass was similar for the STR, SNUF, and 1N1P treatments (26–28 %; Table 4), indicating that the nutrients in both urine-based fertilizers were as efficiently used by ryegrass as those in the reference fertilizer. The constant uptake rate of P from SNUF shows that the P was plant-available throughout the growth experiment (Fig. 2b). Germer et al. (2011) suggested that the P and K requirement of crops can be partially met by human urine, but no previous study has investigated P uptake from stored human urine in detail. The agronomic effectiveness of the P contained in STR (97 %) and SNUF was greater than that of the P contained in other recycling products obtained from wastewater, such as sewage sludge (RAE 62–78 %; Frossard et al. 1996) and sewage sludge ash (RAE 4–88 %; Nanzer et al. 2014).

Our results highlight the importance of using P radioisotope-labeling techniques to quantify plant P uptake from fertilizer relative to soil. With the conventional difference method, Pdff is calculated as the difference in P uptake between a fertilized and an unfertilized treatment. For the 1N1P and STR treatments in the present study, the Pdffmg values obtained with this approach (derived from P uptake in Fig. 1b and using the 1N0P treatment as the unfertilized control) exceeded those obtained with ^33^P radioisotope labeling by about 40 %. This discrepancy suggests that these two treatments increased uptake of soil P by the ryegrass. As mentioned previously, P may have been a plant growth-limiting factor in the 1N0P treatment. It is likely that the P-fertilized treatments allowed greater soil exploration by roots (Morel and Fardeau 1989; Achat et al. 2014b) and therefore took up more soil P, since Pdfs were greater for the P-fertilized (Table 4) than the unfertilized treatment (P uptake in 1N0P; Fig. 1b).

### Effectiveness of N from urine-based fertilizers

The strong linear response to mineral N amendment (0–150 mg N kg^−1^ soil; Fig. S2) revealed that the reference level of 100 mg N kg^−1^ soil applied at sowing to the fertilized treatments was appropriate. The range obtained allowed the plant N recovery of fertilizer (FertNrec) to be compared, including for the lower ^15^N-labeled N dose contained in struvite (23 mg N kg^−1^ soil) compared with SNUF or the water-soluble N reference 1N1P (Table 3). The N concentration in the 0N1P treatment suggested that N was a plant growth-limiting element, since the average N concentration at the first cut (24.5 mg g^−1^) was already below the critical concentration of 28 mg g^−1^ indicated for ryegrass (Bailey et al. 1997), and decreased to 13 mg g^−1^ by the third cut. On the other hand, the N concentration in all treatments receiving the equivalent of 1N or higher was always greater than this critical value.

The similar N recovery (FertNrec, Table 4) of STR, SNUF, and the water-soluble mineral N (1N1P) indicates that the two urine-based fertilizers efficiently supplied N to the ryegrass. Field and greenhouse studies comparing plant N uptake from urine-based and mineral-based fertilizers suggest that human urine N could replace mineral N fertilizers (Germer et al. 2011). The N recovery from ^15^N-labeled urine from monogastrics (Sorensen and Thomsen 2005) and ruminants (Bosshard et al. 2009) is usually reported to be somewhat lower than that from mineral reference fertilizers, a decrease attributed to ammonia losses. The use of ^15^N-labeled synthetic urine-based fertilizer would enable this to be tested under field conditions.

As found for P, higher Ndff values were obtained for the 1N1P and SNUF treatments with the difference method (derived from N uptake in Fig. 1c using the 0N1P treatment as the unfertilized control) than with the isotope method. Similarly to P, N addition probably increased N uptake from the, soil because N deficiency developed rapidly in the non N-fertilized treatment (0N1P).

## Conclusion

The P and N recovery rates and RAE values obtained here showed that the P and N contained in urine-based fertilizers were as readily available to ryegrass growing on a slightly acidic soil as the P and N in water-soluble mineral fertilizers. There is thus technology available to produce effective P and N fertilizers from source-separated human urine. However, further studies are needed on soils with a wider range of chemical properties (e.g., high pH and high P content) and over a range of pedo-climatic conditions in the field. For NUF, further research with real human urine is required to account for the fate of undesired urine compounds, assess the hygiene safety, and determine whether the more complex composition of real urine influences the plant availability of P and N contained in NUF.

## Electronic supplementary material

Below is the link to the electronic supplementary material.

Supplementary material 1 (PDF 356 kb)
