# Supplementary material for: Plant uptake of phosphorus and nitrogen recycled from synthetic source-separated urine
Source: Ambio. 2015 Feb 15;44(Suppl 2):217–27. doi: 10.1007/s13280-014-0616-6 (PMC4329146; doi:10.1007/s13280-014-0616-6)
Supplement: Supplementary file 1 — Supplementary material 1 (PDF 356 kb) [file 13280_2014_616_MOESM1_ESM.pdf]

AMBIO

Electronic Supplementary Material

This supplementary material has not been peer reviewed.

Plant uptake of phosphorus and nitrogen recycled from synthetic source-separated urine

Christophe Bonvin, Bastian Etter, Kai M. Udert, Emmanuel Frossard, Simone Nanzer, Federica Tamburini, Astrid Oberson

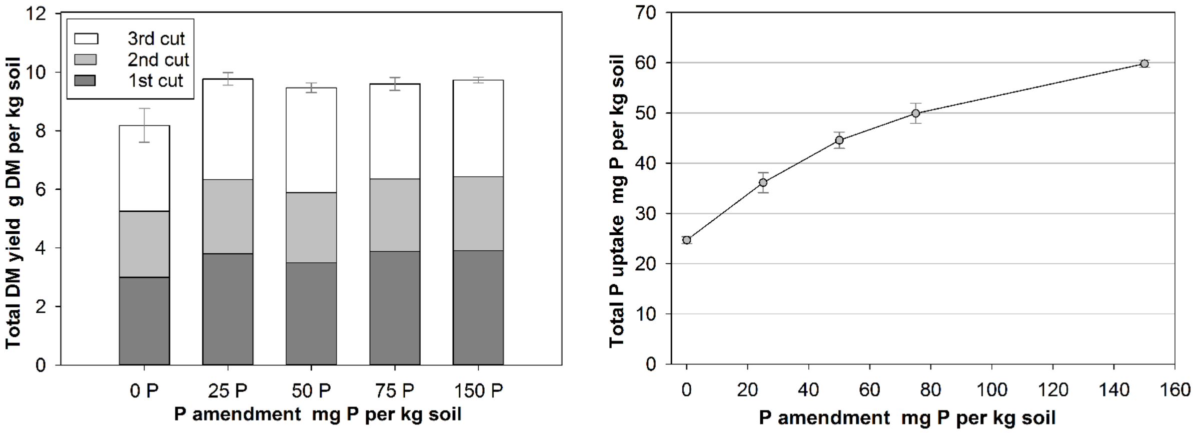

Fig. S1 (Left) Total DM production of Italian ryegrass shoots in treatments with different levels of P fertilisation (P response curve). All treatments received 100 mg N kg^{-1} soil at the start of the experiment and after each cut. (Right) Total P uptake by Italian ryegrass shoots in the same treatments. Vertical bars indicate standard deviations.

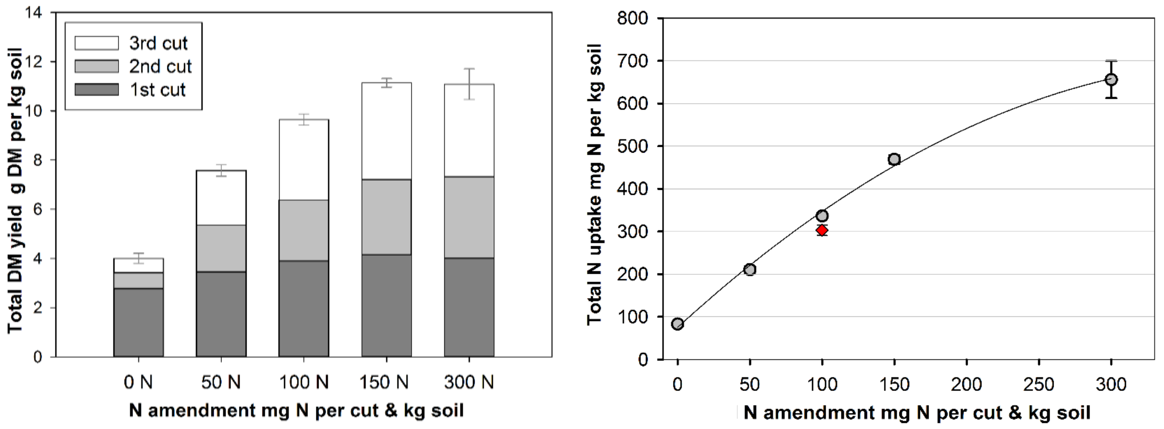

Fig. S2 (Left) Total dry matter (DM) production of Italian ryegrass shoots in treatments with different levels of N fertilisation (N response curve). All treatments apart from 100N (75 mg P) received 50 mg P kg^{-1} soil at the start of the experiment. (Right) Total N uptake of Italian ryegrass shoots of these same treatments. Treatment 1N1P (red diamond) is below the N uptake response curve, confirming the suspicion that it received an erroneous N dose at sowing. Vertical bars indicate standard deviations

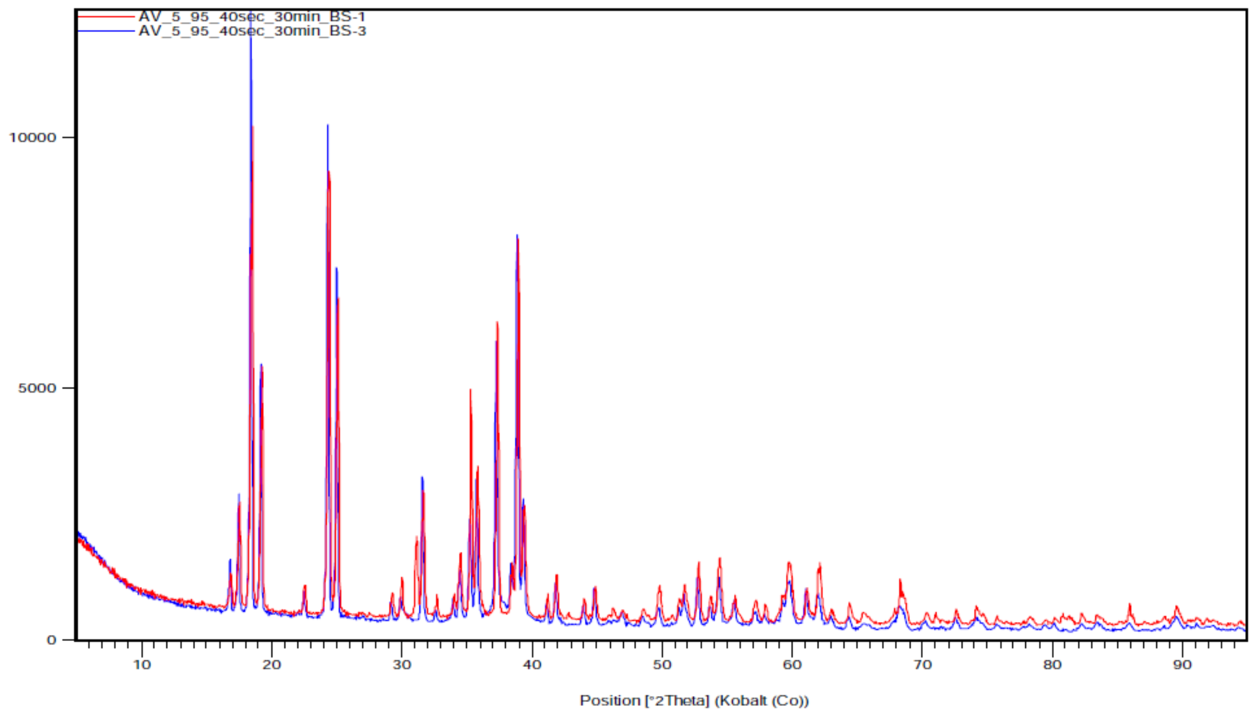

Fig. S3 Qualitative comparison of synthetic struvite used in this experiment (red curve) and struvite processed from real human urine taken from the men's tank at a research institution (blue curve). Matching peak height, width and area suggest that the elemental composition and crystal structure of the two struvites are similar

Table S1 Dry matter (DM) content, total P and N concentration and solubility in water of fertilisers processed from real human urine taken from the urinal tank at a research institution. NUF = nitrified urine fertiliser

**Fertiliser**	**DM content**	**Total N**	**Total P**	**Solubility of elements in H_2O ***	
	%	mg g^{-1}		% of total N	% of total P
Real human urine struvite	88	51	120	2.1	2
Real human urine NUF	94	191	20	93	57

* Water-soluble phosphate, ammonia and nitrate ions determined in product suspensions (2.5 g fertiliser per 250 mL water). Method from European Parliament (2003).
